# Benefits of Expert Online Consultations for the Detection of Pulmonary Embolism and Timely Treatment: Enhancing Telemedicine Technology–A Case Report

**DOI:** 10.1016/j.cjco.2025.05.014

**Published:** 2025-06-02

**Authors:** Masaki Takenaka, Satoshi Yanagisawa, Yukihiko Yoshida, Yasuya Inden, Toyoaki Murohara

**Affiliations:** a Department of Cardiology, Takabari Heart Clinic, Nagoya, Aichi, Japan; b Department of Cardiology, Nagoya University Graduate School of Medicine, Nagoya, Japan; c Department of Advanced Cardiovascular Therapeutics, Nagoya University Graduate School of Medicine, Nagoya, Japan; d Department of Cardiology, Japanese Red Cross Aichi Medical Center, Nagoya Daini Hospital, Nagoya, Japan

**Keywords:** case report, Coronavirus disease, online consultation, pulmonary embolism, telemedicine

**Telemedicine can facilitate communication and patient care for clinicians and patients in remote locations. The coronavirus disease (COVID-19) pandemic necessitated the development of no-contact, remote healthcare solutions, and this innovation has facilitated early diagnosis and treatment in medical care. Herein, we present a case of dyspnea that was detected accurately as a pulmonary embolism, using an innovative approach, through expert online consultations. By leveraging advancements in telemedicine, we were able to detect possible urgent signs of acute pulmonary embolism, and timely treatments could lead to improved patient symptoms and outcomes. We are committed to promoting effective communication to provide high-quality medical care.**

## Case Presentation

A 54-year-old man presented with shortness of breath and was referred to our clinic (Takabari Heart Clinic) for online medical services. He had maintained good health with no significant medical history, and with regular exercise or sports activity until 6 months prior to presentation he contracted COVID-19 during a period of widespread infection. Following his recovery, he opted to continue working at a sedentary job in his home as a precautionary measure against the ongoing pandemic. Several months postinfection, he began to experience shortness of breath during light-intensity running sessions. Owing to his initial suspicion that this symptom could be attributed to reduced physical activity, he monitored his condition closely. However, his shortness of breath persisted, particularly during exertion and while climbing stairs. Due to a demanding workload and a busy schedule, he initially refrained from consulting a physician at a hospital. Instead, the patient decided to use online medical services for professional guidance.

We implemented a standard telemedicine system that is commercially available in Japan (CLINICS, MEDLY Co., Tokyo). This cloud-based system provides patients with a convenient way to schedule appointments and submit their chief complaints and relevant personal information in advance of consultation. This system improves doctors' understanding of patients' conditions before the consultation occurs. After the appointment, payments are processed automatically through a cash card, for added efficiency. Notably, this method does not require the use of specialized cameras for either the physician or the patient; patients need only a personal computer system or a smartphone set up with a standard webcam and microphone, which is convenient for a broad range of patient populations.

During the online consultation, the attending physician conducted a comprehensive evaluation of the patient's current condition and medical history. The patient utilized a standard online camera, which facilitated visual observation of body size and appearance. The assessment indicated that the patient was of normal weight and presented with mild leg edema. The patient's height was 172 cm, and his weight was 62 kg, resulting in a body mass index of 20.1 (Supplemental Fig. S1). The physician suspected development of acute pulmonary embolism in this patient and recommended that he visit a clinic for a face-to-face examination as soon as possible. This assessment was based on the absence of evident coronary risk factors or obvious chest pain, the patient's extended periods of longer and more frequent sitting time following the COVID-19 infection, the relatively rapid progression of shortness of breath that he had not experienced previously, and the lack of orthopnea at night.

**Figure 1:**
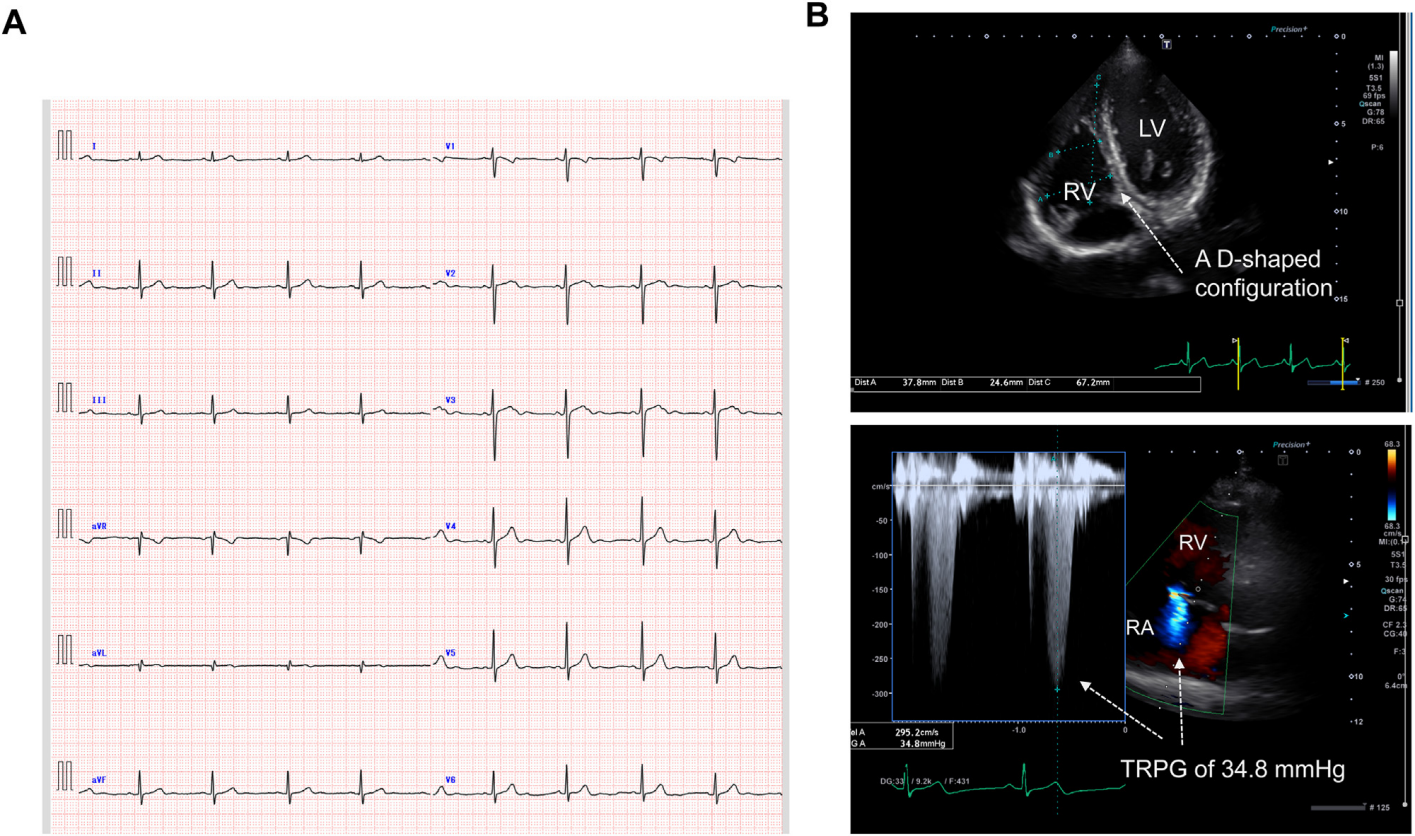
(**A**) A surface 12-lead electrocardiogram and (**B**) transthoracic echocardiography at the first visit for examination. Surface 12-lead electrocardiography in the supine resting position demonstrated sinus rhythm with no significant indicators of right ventricular strain. Transthoracic echocardiography demonstrated a normal left ventricular ejection fraction but revealed right ventricular enlargement, a tricuspid regurgitant pressure gradient (TRPG) of 34.8 mm Hg, and a “D”-shaped configuration in the short-axis image, which suggests the presence of right ventricular hypertrophy. LV, left ventricle; RA, right atrium; RV, right ventricle.

At the clinical consultation, the physical examination revealed mild leg edema, clear breath sounds, and no audible heart murmurs. Upon entry into the consultation room, the following vital signs were documented: blood pressure, 104/76 mm Hg; regular pulse rate, 104 beats per minute; and oxygen saturation level while breathing room air, 93%. In the supine position, surface 12-lead electrocardiography demonstrated a sinus rhythm with no significant indicators of right ventricular strain (Fig. 1A). Chest radiography revealed a cardiothoracic ratio of 43% and did not show any notable enhancement of pulmonary vascular shadows. Transthoracic echocardiography demonstrated a normal left ventricular ejection fraction; but it also revealed right ventricular enlargement, a tricuspid regurgitant pressure gradient (TRPG) of 34.8 mm Hg, and a D-shaped configuration in the short-axis image, which suggests the presence of right ventricular pressure overload (Fig. 1B). Laboratory results indicated elevated levels of fibrin degradation products, at 6.4 μg/mL (normal range, ≤ 4.0 μg/mL) and D-dimer, at 4.9 μg/mL (normal range, ≤ 1.0 μg/mL). Additional coagulation markers included an anti-cardiolipin antibody level of 4.0 U/mL (normal range, ≤ 12.3 μg/mL), a lupus anticoagulant level of 1.0 (normal range, ≤ 1.16), a protein C antigen level of 79.0% (normal range, 64%-146%), and a protein S activity of 85.3% (normal range, 56%-126%).

**Figure 2:**
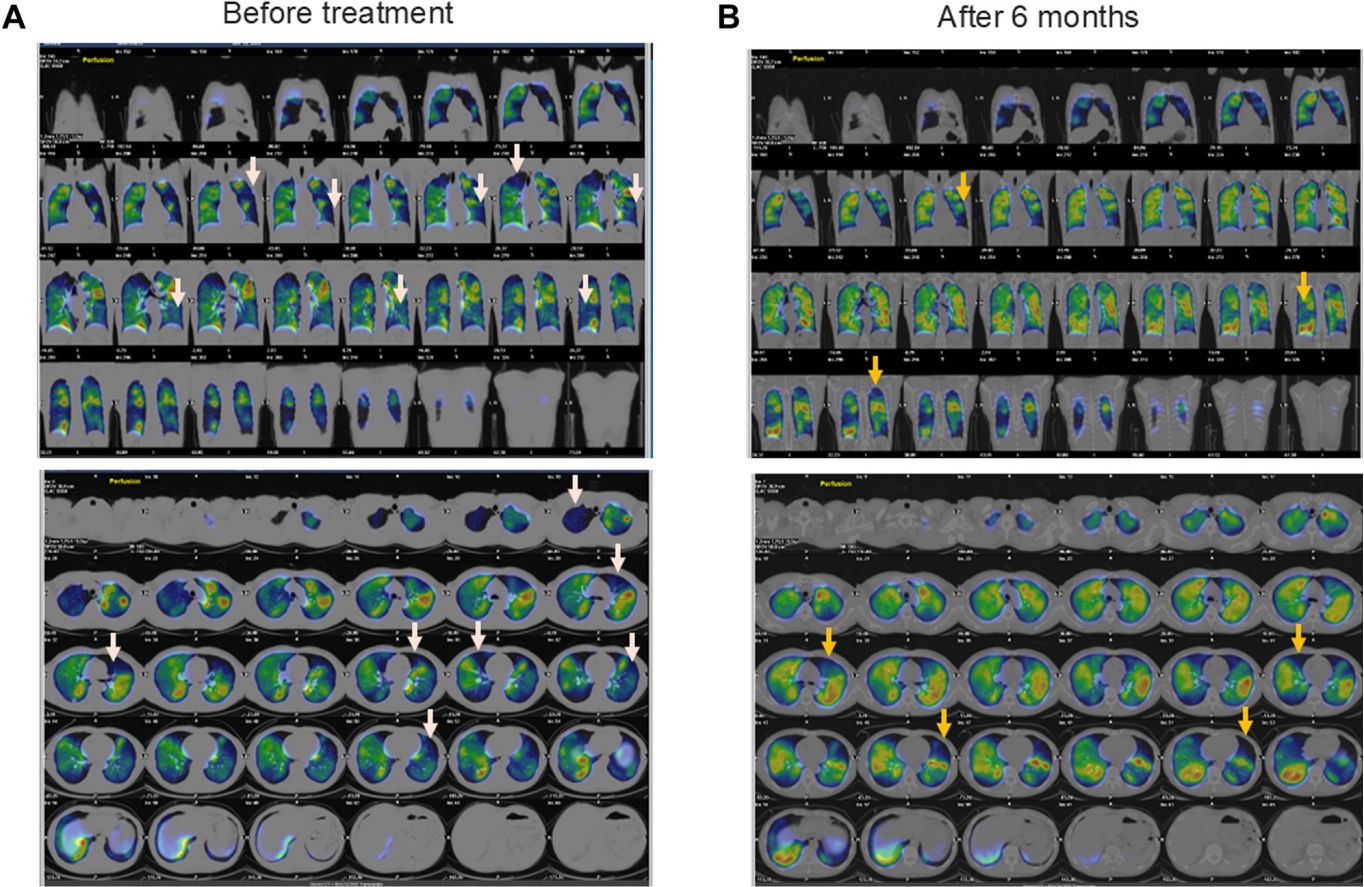
Pulmonary ventilation blood flow scintigraphy (**A**) before treatment and (**B**) after 6 months. Before treatment, the right lung showed defects in the apex, the right middle lobe, and the S6 and S10 regions of the right lower lobe; the left lung showed defects in the left upper segment and S8 region of the left lower lobe. After 6 months, significant improvement had occurred in both the pulmonary apex and the right S10 area. However, defects were still present in the right S2 and S6 areas, the right middle lobe, the left S1+2 and S9 areas, and the tongue area, although the severity of these defects had decreased. The **light gray** and **yellow arrows** indicate the defect area on pulmonary ventilation blood flow scintigraphy.

Contrast-enhanced computed tomography was not performed because of allergy to previous use of contrast media. Subsequent ventilation pulmonary blood flow scintigraphy revealed a bilateral patchy accumulation. Specifically, the right lung showed defects in the apex, right middle lobe, and S6 and S10 regions of the right lower lobe; the left lung showed defects in the left upper segment and S8 region of the left lower lobe (Fig. 2A). Following these assessments, a diagnosis of pulmonary embolism was made, and anticoagulation therapy was started promptly with an initial dose of 30 mg rivaroxaban for 3 weeks, followed by a reduced dose of 15 mg for ongoing treatment.

One month after initiating therapy, the patient's symptoms improved remarkably. He no longer experienced shortness of breath during physical activities involving inclines. After 2 months of treatment, he was able to ascend and descend 6 flights of stairs without difficulty. Follow-up pulmonary ventilation blood flow scintigraphy after 6 months revealed a significant improvement in both the pulmonary apex and right S10 area. However, defects were still present in the right S2 and S6 areas, the right middle lobe, the left S1+2 and S9 areas, and the tongue, although the severity of these defects has decreased (Fig. 2B). Transthoracic echocardiography revealed a reduced TRPG of 18 mm Hg, and relief of the right ventricular load.

Advanced treatment with balloon pulmonary angioplasty on the residual lesions was also recommended to the patient, to improve blood flow. However, the patient prefers to take a “wait-and-see” approach, as his shortness of breath currently is improving and the clinical signs of right ventricular stress have decreased. The patient is monitored at the outpatient clinic by regular pulmonary perfusion scintigraphy, echocardiography, and blood sampling.

## Discussion

The present case study demonstrates the utility of online telemedicine in detecting early symptoms of pulmonary embolism, leading to prompt management and effective treatment (Supplemental Fig. S2). This online consultation might have been the only chance to identify his malignant symptoms via a doctor with appropriate expertise, thus allowing for further hospital consultation and actual treatment before the development of severe dyspnea and sudden death. Online telemedicine may provide numerous occasions to facilitate timely diagnosis and underscores the potential for improvement of evolving healthcare delivery models given the current state of information technology.

The COVID-19 pandemic has profoundly affected healthcare systems worldwide.^1^^,^^2^ The pandemic has led to significant advancements in telehealth and remote consultation.^2^^,^^3^ Evidence suggests that diagnostic accuracy improves significantly when a specialist is involved in the medical interview process.^4^ This method of online medical evaluation can be particularly beneficial for underserved communities, areas with limited healthcare infrastructure, and disaster-related medical needs.^5^^,^^6^

Despite recent advancements in diagnostic tools and imaging modalities, a classical tool—medical interviews for patients—is an essential component of the consultation. The interview remains key for the differential diagnosis of possible diseases and for detection of underlying conditions, feelings, and senses of discomfort. Extensive examinations using advanced imaging modalities and laboratory evaluations covering all parameters can sometimes lead to more accurate diagnoses. However, in-depth conversations with patients, for an adequate duration, can uncover numerous key signs and clues to use in approaching a patient’s overall condition and determining what treatment the patient wants from doctors. In this regard, online telemedicine, which utilizes cameras for visualization and allows direct communication with patients, can facilitate an initial assessment to determine whether a patient's condition is urgent. This understanding is a fundamental component of clinical practice and is consistent with the emphasis it has received in traditional medical education.

The clinical course of this patient suggested an acute-on-chronic pulmonary embolism. Long periods spent working in a sitting position may cause the long-term accumulation of small thrombi in the deep veins of the legs and in the pulmonary artery. This accumulation might explain why electrocardiography at rest demonstrated no remarkable signs of right ventricular strain despite the presence of pulmonary embolism. Although right ventricular pressure overload and TRPG on echocardiography decreased significantly after 6 months, direct imaging data on the residual thrombus in the pulmonary artery, such as contrast-enhanced computed tomography, were not available. Therefore, further continuous monitoring is essential for this patient, given the persistent defects observed in ventilation-perfusion scintigraphy posttreatment and the potential risk of progression to chronic thromboembolic pulmonary hypertension.

The present case demonstrates the utility of online telemedicine in detecting pulmonary embolism, leading to prompt management and effective treatment. Online telemedicine is helpful in medical treatment, and in the current advanced state of the healthcare system, and may enhance the quality of care and patient-related prognosis (Video 1, view video online).

**Novel Teaching Points**

• Online telemedicine can enable the detection of malignant signs of the development of pulmonary embolism and prompt treatment in patients who have busy work schedules that prevent consultation in a clinic.
• This type of telemedicine is helpful in medical treatment, as well as in the current advanced state of the healthcare system, and may enhance the quality of care and patient-related prognosis.
• This case highlights the importance of online, in-depth conversations with patients—of an adequate duration and using a camera—as a fundamental component of clinical practice that can uncover numerous key signs that can be used to approach a patient’s condition.

## Supplementary Material

Supplemental Figures

Video 1 Summary of case presentation and clinical course This video illustrates the telemedicine system in action. It was recorded with the necessary consent from the patient during the evaluation process, which occurred after the initiation of treatment.
