# Supplementary material for: Benefits of Expert Online Consultations for the Detection of Pulmonary Embolism and Timely Treatment: Enhancing Telemedicine Technology–A Case Report
Source: CJC Open. 2025 Jun 2;7(8):1093–6. doi: 10.1016/j.cjco.2025.05.014 (PMC12399112; doi:10.1016/j.cjco.2025.05.014)
Supplement: Supplemental Figures [file mmc1.pdf]

Supplemental Material

Takenaka *et al.*: Benefits of Expert Online Consultations for the Detection of Pulmonary Embolism and Timely Treatment: Enhancing Telemedicine Technology–A Case Report

Table of contents

Page 2: Supplemental Figure S1.

Page 3–4: Supplemental Figure S2.

Page 5: Supplemental Figure S3. CARE checklist

Supplemental Figure S1

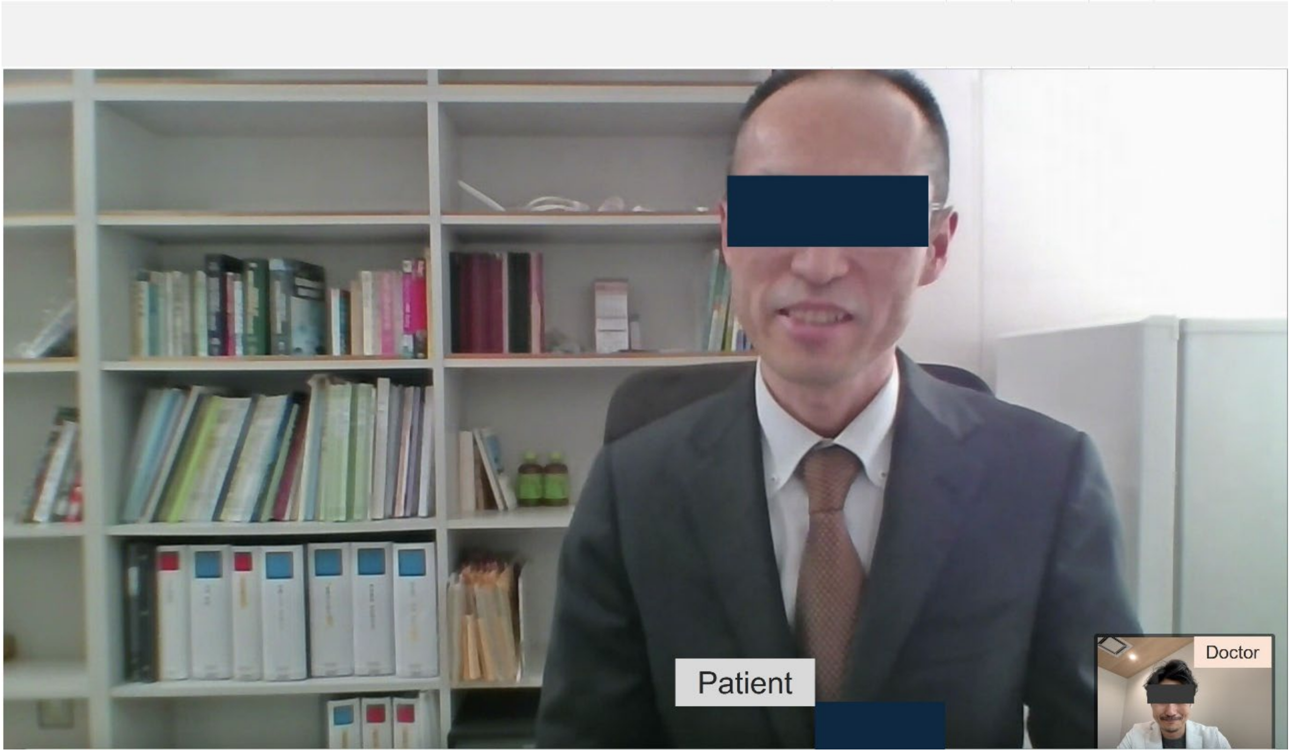

Supplemental Figure S1. This image captures the moment of an online telemedicine consultation using video conferencing between a patient and a doctor. The patients participated from home while the doctor was operating at a clinic. Please note that parts of the individuals in the image were concealed to ensure anonymity, although permission was obtained from the patient to present this image and publish this case report

Supplemental Figure S2: Summary of clinical course

Date	Events
Month 1	A 54-year-old man contracted COVID-19.
Month 2-3	He began to experience shortness of breath during light running sessions, and he was referred to our clinic for online medical services due to demanding work and a busy schedule.
Month 2-3	*The online consultation*<br>The attending physician carefully inquired about the patient's current condition and medical history. The physician suspected the development of an acute pulmonary embolism and recommended that the patient visit a clinic for a face-to-face examination as soon as possible.
Month 3	*The clinical consultation*<br>• Transthoracic echocardiography revealed right ventricular enlargement, a tricuspid regurgitant pressure gradient (TRPG) of 34.8 mmHg, and a D-shaped configuration in the short-axis image.<br>• Laboratory results indicated elevated levels of fibrin degradation products at 6.4 μg/mL and D-dimer at 4.9 μg/mL.<br>• Ventilation pulmonary blood flow scintigraphy revealed a bilateral patchy accumulation.<br>• A diagnosis of pulmonary embolism was made, and anticoagulation therapy was promptly started.
Month 4<br>(1 M after treatment)	He no longer experienced shortness of breath during activities involving inclines.
Month 5<br>(2 M after treatment)	He was able to ascend and descend six flights of stairs without difficulty.
Month 9<br>(6 M after treatment)	• Follow-up pulmonary ventilation blood flow scintigraphy revealed a significant improvement in both the pulmonary apex and right S10 area.<br>• Transthoracic echocardiography revealed a reduced TRPG of 18 mm Hg and relief of the right ventricular load.

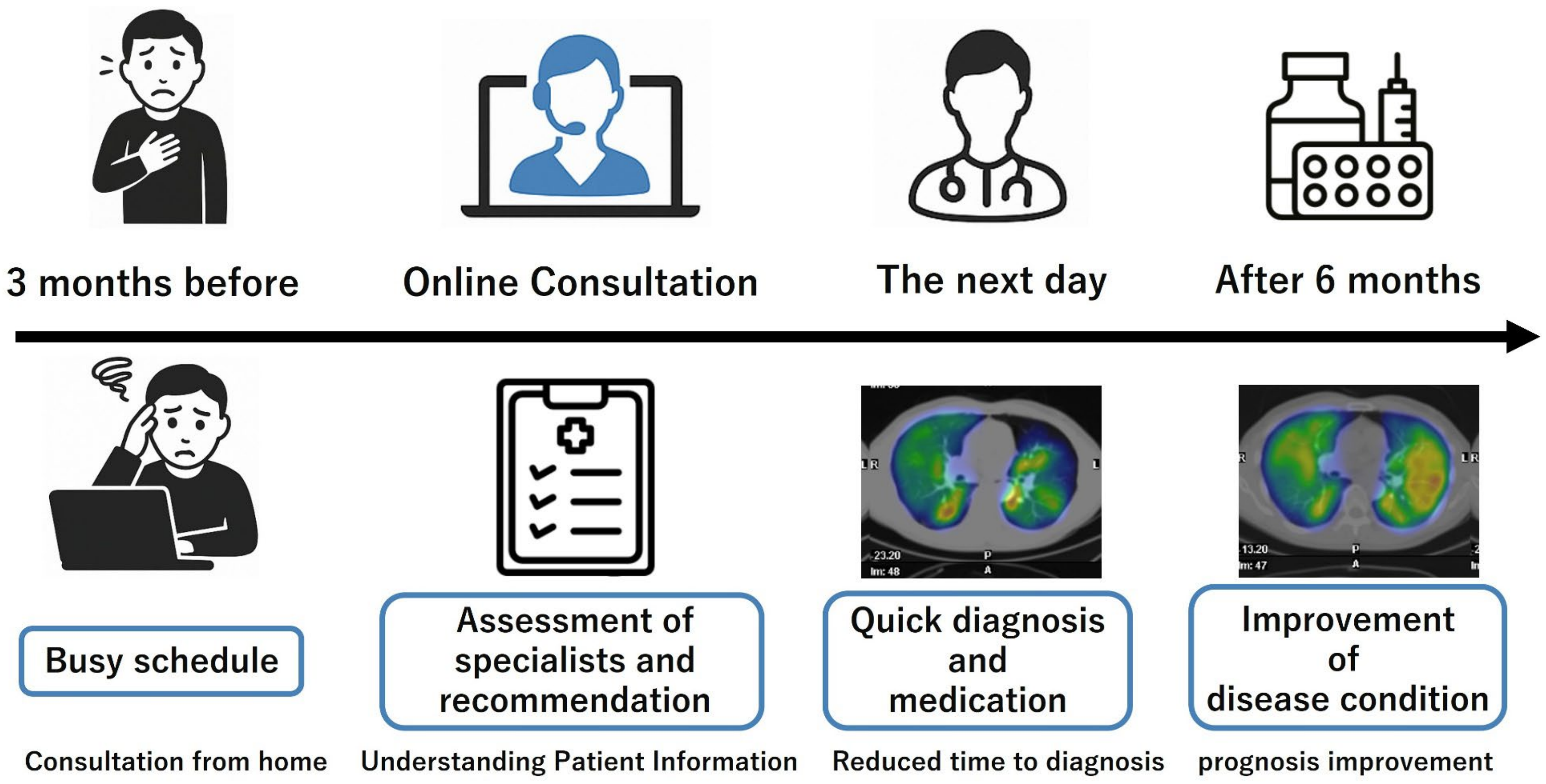
3 months before
Online Consultation
The next day
After 6 months
Busy schedule
Assessment of specialists and recommendation
Quick diagnosis and medication
Improvement of disease condition
Consultation from home
Understanding Patient Information
Reduced time to diagnosis
prognosis improvement

Supplemental Figure S3: CARE checklist

CARE case report guidelines

CARE Checklist of information to include when writing a case report

(cc) BY-NC-ND

Topic	Item	Checklist item description	Reported on Line
Title	1	The diagnosis or intervention of primary focus followed by the words "case report"	lines 1 - 2
Key Words	2	2 to 5 key words that identify diagnoses or interventions in this case report, including "case report"	lines 31 - 32
Abstract (no references)	3a	Introduction: What is unique about this case and what does it add to the scientific literature?	lines 37 - 39
	3b	Main symptoms and/or important clinical findings	lines 39 - 44
	3c	The main diagnoses, therapeutic interventions, and outcomes	lines 37 - 42
	3d	Conclusion—What is the main "take-away" lesson(s) from this case?	lines 39 - 42
Introduction	4	One or two paragraphs summarizing why this case is unique (**may include references**)	lines 34 - 43
Patient Information	5a	De-identified patient specific information.	lines 46 - 47
	5b	Primary concerns and symptoms of the patient.	lines 51 - 54
	5c	Medical, family, and psycho-social history including relevant genetic information	lines 47 - 50
	5d	Relevant past interventions with outcomes	lines 54 - 56
Clinical Findings	6	Describe significant physical examination (PE) and important clinical findings.	lines 77 - 80
Timeline	7	Historical and current information from this episode of care organized as a timeline	lines 47 - 56
Diagnostic Assessment	8a	Diagnostic testing (such as PE, laboratory testing, imaging, surveys).	lines 77 - 97
	8b	Diagnostic challenges (such as access to testing, financial, or cultural)	lines 83 - 100
	8c	Diagnosis (including other diagnoses considered)	lines 97 - 100
	8d	Prognosis (such as staging in oncology) where applicable	N/A
Therapeutic Intervention	9a	Types of therapeutic intervention (such as pharmacologic, surgical, preventive, self-care)	lines 97 - 100
	9b	Administration of therapeutic intervention (such as dosage, strength, duration)	lines 97 - 100
	9c	Changes in therapeutic intervention (with rationale)	N/A
Follow-up and Outcomes	10a	Clinician and patient-assessed outcomes (if available)	lines 101 - 113
	10b	Important follow-up diagnostic and other test results	lines 101 - 108
	10c	Intervention adherence and tolerability (How was this assessed?)	lines 109 - 113
	10d	Adverse and unanticipated events	N/A
Discussion	11a	A scientific discussion of the strengths AND limitations associated with this case report	lines 146 - 149
	11b	Discussion of the relevant medical literature **with references**.	lines 188 - 205
	11c	The scientific rationale for any conclusions (including assessment of possible causes)	lines 130 - 141
	11d	The primary "take-away" lessons of this case report (without references) in a one paragraph conclusion	lines 153 - 157
Patient Perspective	12	The patient should share their perspective in one to two paragraphs on the treatment(s) they received	lines 97 - 100
Informed Consent	13	Did the patient give informed consent? Please provide if requested	Yes ☑ No ☐
